# Three-Dimensional Printing of Drug-Eluting Implantable PLGA Scaffolds for Bone Regeneration

**DOI:** 10.3390/bioengineering11030259

**Published:** 2024-03-06

**Authors:** Manjusha Annaji, Nur Mita, Ishwor Poudel, Sai H. S. Boddu, Oladiran Fasina, R. Jayachandra Babu

**Affiliations:** 1 Department of Drug Discovery and Development, Harrison College of Pharmacy, Auburn University, Auburn, AL 36849, USA; mza0153@auburn.edu (M.A.); or mita@farmasi.unmul.ac.id (N.M.); izp0018@auburn.edu (I.P.); 2 Faculty of Pharmacy, Mulawarman University, Samarinda, Kalimantan Timur 75119, Indonesia; 3 Department of Pharmaceutical Sciences, College of Pharmacy and Health Sciences, Ajman University, Ajman P.O. Box 346, United Arab Emirates; s.boddu@ajman.ac.ae; 4 Center of Medical and Bio-Allied Health Sciences Research, Ajman University, Ajman P.O. Box 346, United Arab Emirates; 5 Department of Biosystems Engineering, Samuel Ginn College of Engineering, Auburn University, Auburn, AL 36849, USA; fasinoo@auburn.edu

**Keywords:** 3D printing, ketoprofen, PLGA scaffolds, sustained release, thermoplastic extrusion

## Abstract

Despite rapid progress in tissue engineering, the repair and regeneration of bone defects remains challenging, especially for non-homogenous and complicated defects. We have developed and characterized biodegradable drug-eluting scaffolds for bone regeneration utilizing direct powder extrusion-based three-dimensional (3D) printing techniques. The PLGA scaffolds were fabricated using poly (lactic-co-glycolic acid) (PLGA) with inherent viscosities of 0.2 dl/g and 0.4 dl/g and ketoprofen. The effect of parameters such as the infill, geometry, and wall thickness of the drug carrier on the release kinetics of ketoprofen was studied. The release studies revealed that infill density significantly impacts the release performance, where 10% infill showed faster and almost complete release of the drug, whereas 50% infill demonstrated a sustained release. The Korsmeyer–Peppas model showed the best fit for release data irrespective of the PLGA molecular weight and infill density. It was demonstrated that printing parameters such as infill density, scaffold wall thickness, and geometry played an important role in controlling the release and, therefore, in designing customized drug-eluting scaffolds for bone regeneration.

## 1. Introduction

Tissue engineering is a rapidly emerging field that produces temporary constructs for the regeneration, restoration, and enhancement of the function of cells and tissues in the body [1,2]. Some of the clinical applications for bone regeneration include loss of bone following skeletal trauma, bone defects that occur after primary tumor resection, and trabecular voids that were created due to osteoporotic insufficiency fractures [3,4]. In this regard, various approaches have been implemented, such as implanting the cells isolated from the humans into the defect areas, delivering drugs and proteins that can induce tissue growth and regeneration, and utilizing three-dimensional (3D)-printed porous polymeric scaffolds. Among all, the 3D printing of scaffolds is more prominent due to its ability to act as a substrate that can promote cell adhesion and proliferation. These biomaterials have higher osteointegration capacity with minimal host response at the implantation site, thus promoting tissue regeneration. Moreover, due to the biodegradable nature of polymeric scaffolds, the material will be completely integrated into the host tissue without needing revision surgeries. Many natural (hyaluronic acid, carboxymethylcellulose, chitosan, and collagen) and synthetic (polylactic acid, polylactide-co-glycolide (PLGA), and polycaprolactone) polymers have been widely utilized. PLGA is one of the most commonly studied polymers due to its safety, biocompatibility, and biodegradability [5,6]. It has been approved by the FDA for manufacturing implantable scaffolds [7]. Several studies in the past have demonstrated the biocompatibility of PLGA-based implants. In a study by Liu CG et al., PLGA scaffolds prepared by FDM-based 3D printing exhibited excellent biocompatibility, thereby promoting the cell growth, proliferation, and distribution of preosteoblasts in the scaffolds [8]. Similarly, in another study by Yang et al., 3D-printed PLGA/HA scaffolds showed good biocompatibility and osteogenic activity in rats [9].

These artificial constructs can be implanted into the body to promote faster regeneration, either with or without the cells and/or growth factors. Traditional drug administration through the systemic route has several limitations, such as the need for the administration of higher doses to achieve therapeutic concentrations and a lack of target specificity leading to adverse drug reactions. To overcome such challenges, drug-eluting artificial constructs have been developed. These drug-eluting constructs can provide sustained and targeted delivery of drugs directly at the implantation site, thereby promoting bone healing and regeneration and thus minimizing systemic toxicity [10]. The implantation of these artificial constructs often leads to pain and inflammation at the implantation site. Hence, it is essential to deliver anti-inflammatory and immunosuppressive drugs to promote tissue regeneration. Some of the previous reports have developed ketoprofen-eluting scaffolds for bone regeneration. In a study by Raafat et al., ketoprofen was utilized as a model anti-inflammatory drug to evaluate the bioglass reinforced composite scaffold potential as a drug carrier [11]. Similarly, in a study by Prabaharan et al., poly(L-lactic acid)–chitosan hybrid scaffolds were prepared utilizing ketoprofen as a model drug for studying the effectiveness of the scaffold as a drug delivery system in tissue engineering applications [12]. In the current study, ketoprofen was chosen as a model anti-inflammatory and anti-rheumatic drug to evaluate the potential of PLGA scaffolds as drug carriers. It is also highly critical to ensure that the carrier has the potential to fully retain the drug before implantation and provide controlled release upon implantation. In addition, the scaffolds should also meet the criteria of injectable products, such as sterility and apyrogenicity [13].

However, it is critical to fabricate porous architectures with sufficient mechanical properties to enhance osteointegration and to improve cell migration [14]. Various strategies have been explored in the past for the fabrication of scaffolds, such as particle leaching [15] and phase separation [16]. However, due to a lack of proper control over the infill and wall thickness, the pores were randomly created with poor interconnections and thus did not provide sufficient strength for utilization in the bone defects [17]. Therefore, to fabricate structures with higher control over infill, geometry, and wall thickness, three-dimensional (3D) printing techniques such as stereolithography [18], fused deposition modeling (FDM) [19], and selective laser sintering (SLS) [20] have been utilized. The advantage of 3D printing is its ability to design structures using computer-aided design (CAD) to fabricate structures with the desired properties that can exactly mimic the anatomy and physiology of human bone [21]. The incorporation of 3D printing to prepare bone substitutes allows one to improve osteoconduction through an optimized infill density [22], simultaneously controlling the mechanical properties of the scaffolds by modulating the wall thickness [23]. All the previously utilized 3D printing technologies require either the prefabrication of filaments or the usage of solvents and binders for their fabrication [24,25]. This might lead to an increased risk of residual solvents in the implants. Hence, the direct melt extrusion method was utilized in the current study to fabricate PLGA scaffolds without the need for the prefabrication of filaments and the usage of additional solvents.

Modulating the release profile is one of the major advantages of 3D-printed technology. Among various printing parameters, infill density is the most unique parameter and controls the percentage of material to be printed in each layer. This parameter can be controlled during the slicing step of the printing process. In some previous studies, the effect of infill percentage on the mechanical properties of 3D-printed PLA structures was investigated [26,27,28]. It was observed that the infill density, extrusion temperature, infill pattern, and layer thickness had a significant role in modulating the mechanical strength and release profile of the drug [29]. Structures with higher tensile strength were obtained by utilizing a combination of a high infill percentage, hexagonal infill pattern, lower thickness of the layers, and higher temperature [29]. Similarly, in a study by Fanous et al., Eudragit-based printlets were 3D-printed with different infill densities (65 to 100%) in order to modulate the release profile. It was observed that printlets with 65% infill showed faster release compared to those with 100% infill [30]. Though the use of 3D-printed scaffolds is a great alternative for the regenerative medicine community, the evaluation of critical parameters that contribute to successful design for host integration remains a non-standardized process. In the case of PLGA-based scaffolds, the accessible literature essentially deals with the relationship between mechanical properties, in vitro release, and geometrical parameters (infill density, geometry, and wall thickness) separately and only very limited reports study them together. Hence, this study proposes a combined set of scaffold parameters that can be used to modulate the mechanical properties and drug release, thus enhancing the host integration into vascularized tissues. This approach can be applied to a broad range of tissue engineered products, from conception through development. We aim to fabricate and characterize 3D-printed implantable PLGA scaffolds for promoting bone regeneration using ketoprofen as a model drug. Various parameters, such as the infill, geometry, and wall thickness of the drug carrier, can directly affect the release kinetics of the drug over time [31]. Therefore, the effect of such parameters on the in vitro drug release was considered for investigation.

## 2. Materials and Methods

### 2.1. Materials

USP grade ketoprofen (Lot No. 1506190117) was obtained from Letco Medical (Decatur, AL, USA); PLGA 50:50 with inherent viscosities 0.2 dl/g and 0.4 dl/g acid-terminated were purchased from Polysciences Inc. (Warrington, PA, USA). All other analytical solvents of HPLC grade were procured from VWR International (Suwannee, GA, USA), and Milli Q water was obtained from an in-house Millipore water purification system.

### 2.2. Determination of Saturation Solubility

Determination of saturation solubility of ketoprofen was carried out in various pharmaceutical solvents (water, PBS pH 7.4, PBS/ethanol (9:1% v/v), PBS/ethanol (8:2% v/v), and PBS/methanol (9:1% v/v)) to select appropriate release media for the in vitro release studies. An excess amount of drug was added to 1 mL of solvent, and the resulting mixture was shaken reciprocally at 25 °C for 24 h, followed by equilibration and centrifugation at 10,000 rpm for 10 min. The supernatant was filtered through a membrane filter (0.22 μm), and appropriate dilutions were made in the mobile phase and analyzed via HPLC.

### 2.3. Three-Dimensional Fabrication of Implantable PLGA Scaffolds

The implantable scaffolds were printed using a 3D Bioprinter (Bio X, Cellink, Gothenburg, Sweden), utilizing a direct powder extrusion-based 3D printing technology. PLGA 50:50 acid-terminated with two different inherent viscosities, 0.2 dl/g and 0.4 dl/g, was used in this study. Implants were designed using computer-aided design (CAD) software (SolidWorks 2020, Dassault Systemes SolidWorks Corp., Waltham, MA, USA) and sliced using HeartOS 1.4 Cellink software to enable 3D printing of implantable scaffolds. PLGA and ketoprofen at a 9:1 ratio were thoroughly blended and loaded into a stainless-steel cartridge of the thermoplastic print head. A stainless-steel nozzle with an inner diameter of 0.4 mm was utilized for extruding the molten blend. The blend inside the cartridge was heated for 20 min to ensure the blend was melted completely to achieve homogenous drug distribution. Printing was performed at an extrusion pressure of 200 kPa and 180 kPa and a printing temperature of 90 °C and 120 °C for PLGA with an inherent viscosity of 0.2 and 0.4 dl/g, respectively. Various printing parameters, such as printing speed, temperature, and pressure, were optimized based on the trials conducted. The build plate temperature was maintained at 25 °C, and the printing speed was kept constant at 2 mm/s for PLGAs with different inherent viscosities. Scaffolds with varying infill densities (10, 20, 30, 40, and 50%) were printed. Blue painter’s tape was used on the print bed to allow for greater adhesion of the first layer and easy removal of the structures after printing. To evaluate the effect of geometry on the in vitro release, two different geometries, such as a cylinder and a square, were printed. The effect of scaffold wall thickness (1 and 3 mm) on the in vitro drug release was studied. Twelve scaffolds for each PLGA viscosity were printed, and all the scaffolds were stored in a desiccator at room temperature until further characterization.

### 2.4. Characterization of Scaffold Dimensions

The length, width, and thickness of the scaffolds were determined using Vernier calipers (ASX, Mitutoyo Corporation, Kanagawa, Japan). The weight of the six scaffolds was determined, and all the values were reported as mean ± standard deviation.

### 2.5. Quantitative Analysis of Ketoprofen Content

Six scaffolds per batch were accurately weighed and placed in a separate screw-capped vial containing 10 mL of acetonitrile and sonicated for 1 h. The solutions were left overnight at room temperature to allow for complete dissolution of the drug and the polymer. The solutions were centrifuged at 10,000 rpm for 10 min, followed by filtration and dilution with acetonitrile. The samples were analyzed by the HPLC method, as described below. The values were reported as mean ± standard deviation.

The HPLC system consisted of a UV detector and a separation module (Alliance e2695, Waters Corporation, Milford, MA, USA). A reverse phase C18 column of 150 × 4.6 mm dimensions with 5 μm particles (Phenomenex, Torrance, CA, USA) was utilized. The mobile phase consisted of methanol and water (0.1% phosphoric acid) (75:25), which was run at a flow rate of 1 mL/min. The detection was carried out at a wavelength of 254 nm.

### 2.6. Determination of Surface Roughness and Surface Morphology of Scaffolds

Surface roughness of the 3D-printed scaffolds was determined using a Keyence VHX-6000 digital microscope. The measurements were carried out at 100× and 200× magnifications.

The surface morphology of placebo and ketoprofen-eluting scaffolds at 10, 30, and 50% infill densities was visualized using a high-resolution focused ion beam-scanning electron microscope (FIB-SEM ZEISS Crossbeam 550, Carl ZEISS Microscopy, Oberkochen, Germany) at an accelerating voltage of 10 kV. Scaffolds were mounted on the stubs using double adhesive tape, and the images were captured at 13× and 100× magnifications.

### 2.7. Mechanical Testing of Scaffolds

The compressive strength of the as-prepared 3D-printed placebo and ketoprofen-loaded scaffolds was determined using a TA-HDi Texture Analyzer (Texture Technologies Corp, Hamilton, MA, USA). Placebo and ketoprofen-eluting scaffolds (n = 3 for each infill density) were pressed against a stainless-steel surface with a constant deformation rate of 0.1 mm/s, and the compressive strength was indicated by a sudden drop in the applied force. The values were presented as mean ± standard error of the mean.

### 2.8. Differential Scanning Calorimetry

Differential scanning calorimetry (TA Instruments Model Q200, New Castle, DE, USA) with two-stage cooling was ramped from 10 to 200 °C at 10 °C/min. Individual specimens were weighed (2–5 mg) using a calibrated balance and placed in sealed aluminum pans (DSC consumables incorporated, Austin, MN, USA). DSC of the pure polymer, ketoprofen, ketoprofen–PLGA physical mixture, and ketoprofen-eluting scaffolds was performed to determine the glass transition as a function of temperature to confirm if there were any changes in drug solid-state behavior after being incorporated in the PLGA matrix during fabrication. Thermograms were analyzed using Universal Analysis v5.5.24 Software. The data were presented as a plot of temperature (°C) vs. heat flow (arbitrary units).

### 2.9. Static Contact Angle Measurement

The hydrophilicity of the implantable scaffold surfaces was determined by measuring the water contact angle (θ) between the surface of the scaffold and the contour of the water droplet. In this experiment, a fully computer-controlled contact angle meter from KSV Instruments Ltd. (Helsinki, Finland) containing a CAM 200 video camera was used to measure the wettability of the surface. The parameters were set at a measuring speed of 32 data points/s, a droplet volume of 1.5 μL, and a temperature of 23 °C. The contact angle was measured for a period of 10 s. Three different locations of the same sample were measured to determine the mean contact angle, and the values were presented as mean ± standard deviation.

### 2.10. In Vitro Release Studies

In vitro, release tests were carried out by placing the scaffolds in 10 mL of PBS pH 7.4 and agitating at 37 °C at 50 rpm. The vials were shaken in a sealed condition to prevent the evaporation of release media. To maintain sink conditions, the samples (1 mL) were withdrawn at predetermined time intervals and replaced with the same volume of fresh media. The release samples were analyzed by HPLC, as described earlier. The cumulative drug concentration was plotted against the release time. The effect of infill density, geometry, and wall thickness on the in vitro release of ketoprofen was studied.

### 2.11. Drug Release Kinetics

The mechanism of drug release was studied using various mathematical models, such as zero, first, Higuchi, Hixson–Crowell, and Korsmeyer–Peppas models. The best-fit models were selected based on the R^2^ value for each group.

### 2.12. Statistical Analysis

All the experiments were performed in triplicate, and the statistical analyses were carried out using GraphPad Prism (Prism 9, GraphPad Software, San Diego, CA, USA) software. Student *t*-test was performed to determine the significant differences between the weight vs. infill and drug loading vs. infill printed with two different PLGAs. The level of significance was accepted at *p* < 0.05.

## 3. Results and Discussion

### 3.1. Saturation Solubility of Ketoprofen

Table S1 shows the solubility of ketoprofen in various pharmaceutical solvents. Ketoprofen has very low solubility in water (0.135 ± 0.38 mg/mL). However, in PBS pH 7.4, PBS pH 7.4 + ethanol (9:1% v/v), and PBS pH 7.4 + ethanol (8:2% v/v), its solubility is 0.404 ± 0.21 mg/mL, 0.405 ± 0.16 mg/mL, and 0.415 ± 0.04 mg/mL, respectively. Solubility was not significantly increased when ethanol was incorporated into the release media. Moreover, ketoprofen effectively maintained sink conditions in PBS pH 7.4 during the preliminary in vitro trials. Therefore, PBS pH 7.4 was utilized for the in vitro release studies.

### 3.2. Three-Dimensional Printing of Porous PLGA Scaffolds

Drug-eluting scaffolds are a combination of potential carriers and drugs for bone repair and regeneration. Such interactions are essential for enhancing the functionality and mechanical properties of drug-eluting scaffolds. Controlling the printing parameters, such as the infill, wall thickness, and geometry, is critical to control the mechanical strength as well as the release and degradation of a printed structure [32]. There are very limited studies reporting the effect of printing parameters on the in vitro release of drugs. We have successfully fabricated 50:50 PLGA scaffolds with designed infill densities and geometries using the direct powder extrusion technique to examine the effect of printing parameters on the in vitro release of ketoprofen. The physical properties of a PLGA 50:50 acid-terminated polymer are provided in Table S2. Some of the previous reports have utilized ketoprofen as a model anti-inflammatory and analgesic drug for evaluating composite scaffolds [11]. The data obtained in this study can be utilized as a starting material to design porous implantable scaffolds without the need to modify the composition. Drug release can be tailored to the specific needs of the patient, thus providing customized drug-eluting structures for bone regeneration and repair.

Based on the initial screening of the melting and degradation temperatures, the printing temperature was optimized to 90 and 120 °C, respectively, for PLGAs with 0.2 and 0.4 dl/g inherent viscosities. As shown in Table 1, the printing temperatures were lowered so that the drug was not subjected to higher thermal stresses. After evaluating the compatibility, it was confirmed that the drug and the polymer were highly likely to be miscible and, therefore, form a solid dispersion. For the preparation of implants with medium-molecular-weight PLGA, it was observed that the printing temperature increased from 90 to 120 °C due to the higher viscosity of the polymer. The printing speed and pressure were constantly monitored to maintain the same diameter of the nozzle for printing scaffolds. As an alternative, the printing temperature was slightly increased, but not higher than 120 °C, even though the drug was confirmed to be thermostable at higher temperatures (150 °C). Finally, post-printing, the drug content and content uniformity of the batches were investigated, and the percentage assay was found to be within 95–105% (Table 2).

### 3.3. Surface Morphology and Surface Roughness

**Figure 1:**
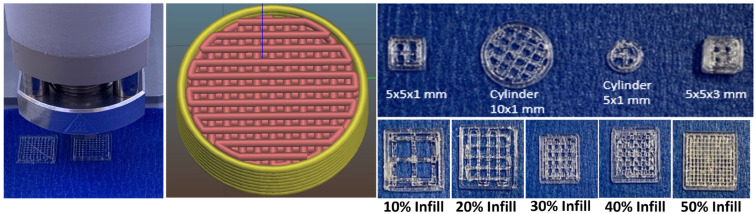
Images of 3D-printed ketoprofen-eluting PLGA scaffolds printed with different infill densities and geometries. The X and Y coordinates are represented by green and blue lines respectively.

**Figure 2:**
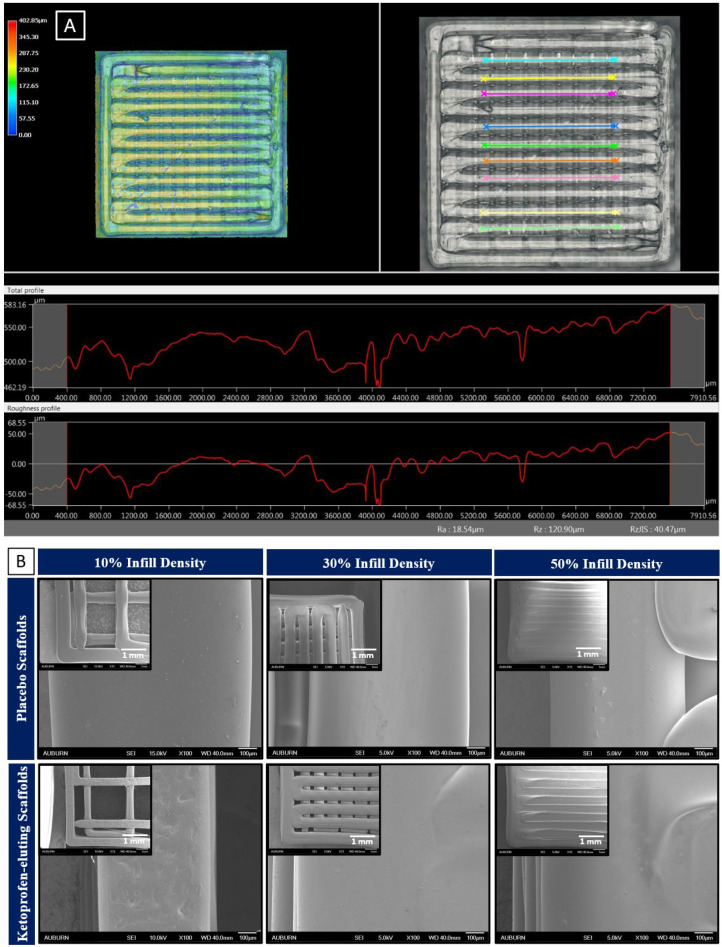
(**A**) Average surface roughness (Ra) measured by Keyence digital microscope (ketoprofen-eluting scaffold printed with PLGA 0.4 dl/g acid-terminated); (**B**) FIB-SEM images of placebo and ketoprofen-eluting scaffolds at 13× and 100× magnifications.

The ketoprofen scaffolds showed variation in structures with different infill densities (Figure 1). Implants resembling porous structures were 3D-printed and tested for the in vitro release studies. Keyence high-resolution images of 3D-printed scaffolds revealed a rough surface with uniformly dispersed drugs throughout the scaffold. The surface roughness of the ketoprofen-eluting scaffold was around 18 μm (Figure 2A). Three-dimensional printing has the ability to control the surface roughness of printed structures. By adjusting various printing and process parameters, such as layer thickness, printing temperature, and speed, the surface roughness of the scaffolds can be modulated [33,34]. Figure 2B shows SEM images of the 3D-printed scaffolds with their porous structure. All the scaffolds showed a smooth surface with well-aligned straight threads printed layer-by-layer in horizontal and vertical directions with perpendicular crossings. There was a decrease in the proximity of the lines as the infill density increased from 10 to 50%, which also corresponds to a reduction in the pore size. PLGA scaffolds showed a smoother surface, whereas ketoprofen-eluting scaffolds displayed a rough surface. Similar findings were shown in previous studies, where the incorporation of the drug reduced the smoothness of the scaffold surface [35]. Additionally, the microstructure of the scaffolds determines the cell penetration efficiency into the scaffold [36]. Scaffolds with a high degree of porosity (~90%) and an interconnected pore network are considered to be ideal for cell interaction and integration with the host tissue [37]. Previous studies with PLGA scaffolds have evaluated the porosity, pore interconnectivity, and surface area. In a study by Dorati et al., the porosity of 3D-printed PLGA scaffolds was evaluated. Scaffolds with high porosity (83.8–89.4%) and interconnected networks were obtained. Very small pores prevented the cells from penetrating into the scaffolds, whereas larger pores prevented cell attachment due to decreased surface area [38].

**Figure 3:**
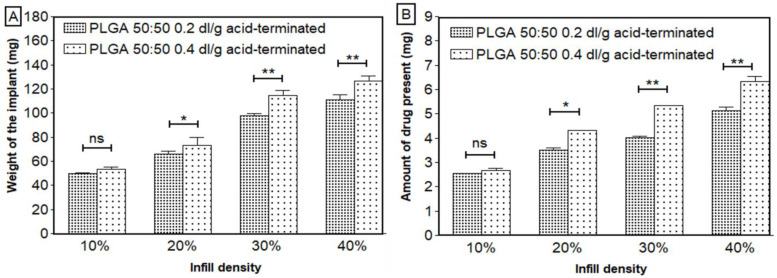
(**A**) Weight and (**B**) amount of ketoprofen present in the scaffolds with respect to infill densities (n = 3, mean ± SD). * *p* < 0.05, ** *p* < 0.01, ns—not significant.

In this study, we showed that the dose of ketoprofen can be adjusted by varying the infill density. As the size of the scaffold did not change, the weight and ketoprofen dose increased with higher infill densities (Figure 3). Figure 3A shows that the weight and infill density increased linearly. The drug distribution was homogenous, as confirmed by the data shown in Figure 3B, where there was a linear relation between the amount of ketoprofen loading and infill density with an R^2^ of 0.9935 and 0.9952 for structures printed with PLGA 0.2 dl/g and 0.4 dl/g, respectively, and at each infill density, the RSD was no more than 5%. A similar pattern was observed previously, where a linear correlation was obtained between the infill and weight of 3D-printed tablets [39]. Similarly, in a study by Korte et al., theophylline-loaded 3D-printed network structures showed a linear correlation of their weight and doses with infill densities. Dose adaptation and the estimation of drug release from the sustained release drug delivery system were performed by changing the infill densities of the 3D-printed network structures [40].

### 3.4. Mechanical Strength of Scaffolds

To study the mechanical properties of the 3D-printed scaffolds, their compression strength was evaluated. Scaffolds with poor mechanical strength are likely to break during implantation; therefore, scaffolds should have sufficient strength to withstand insertion for the duration of drug release. If the scaffold breaks or cracks after implantation, it might cause the burst release of the drug, leading to undesirable side effects for the patient. The compression strength of the placebo and ketoprofen-loaded implants is shown in Figure S1. The addition of ketoprofen did not lead to a statistical change in the compression strength compared to placebo scaffolds. However, an increase in the infill density increased the compression strength for both the placebo and ketoprofen-loaded scaffolds. A larger force was required to break the ketoprofen scaffolds with 50% infill density (14.10 ± 0.25 N) compared to scaffolds with 10% infill (12.53 ± 0.05 N). This might be due to an increase in the density of the scaffolds with an increase in the filling degree. Similar findings were obtained previously for PLGA-based porous scaffolds [8,41]. Overall, an increase in the infill density improved the mechanical properties of the 3D-printed scaffolds.

### 3.5. Thermal Analysis

**Figure 4:**
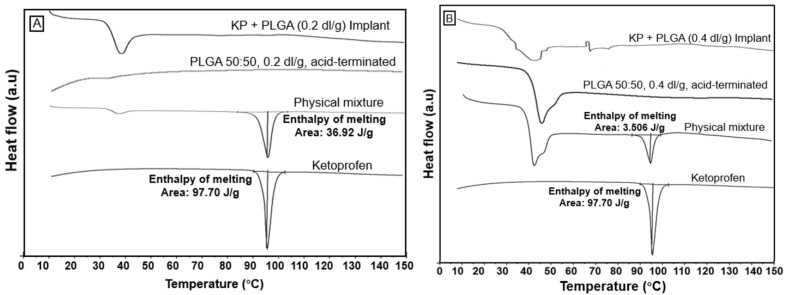
DSC curves of pure ketoprofen, PLGA, physical mixture, and ketoprofen-eluting PLGA scaffolds of (**A**) PLGA 0.2 dl/g acid-terminated and (**B**) PLGA 0.4 dl/g acid-terminated. The vertical line in the figure represent the melting temperature of ketoprofen.

As shown in Figure 4, ketoprofen exhibited a thermal transition at 96 °C, i.e., the melting temperature of the drug. The glass transition temperature (Tg) for PLGA 0.2 dl/g and 0.4 dl/g was observed between 30 and 50 °C as a function of polymer composition. The physical mixture depicts the melting peak of both ketoprofen and PLGA, which is similar to their pure forms, indicating the crystalline nature of the drug. The melting enthalpy of pure ketoprofen was 97.70 J/g, whereas the melting enthalpy of PLGA + KP 50:50, 0.2 dl/g acid-terminated was 36.92 J/g and the melting enthalpy of PLGA + KP 50:50, 0.4 dl/g acid-terminated was 3.506 J/g. However, in the case of ketoprofen-eluting PLGA scaffolds (0.2 dl/g), the endothermic peak at the melting point of ketoprofen was absent, confirming the change in the solid state and complete amorphization of the drug in the matrix. Similarly, the DSC curve of KP + PLGA (0.4 dl/g) in Figure 4 shows that the melting endotherm of ketoprofen was shifted to the left and there is no drug peak near its melting point. This demonstrates that there is a change in the solid state of the drug from crystalline to the amorphous state and partial amorphization of the drug in the matrix.

### 3.6. Contact Angle Measurements

**Figure 5:**
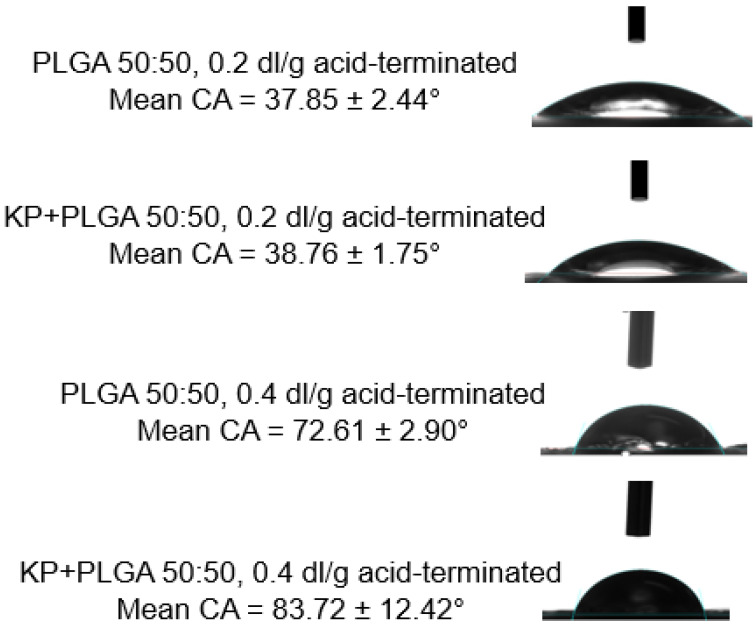
Contact angle measurement of different ketoprofen-eluting scaffolds (n = 3, mean ± SD).

Surface wettability is an important parameter in tissue engineering. Hydrophilic surfaces are highly favorable for cell adhesion and proliferation [42]. The surface wettability of ketoprofen scaffolds was determined by measuring the contact angle between the PLGA scaffold and the water droplet. A change in the contact angle values between the placebo and ketoprofen-loaded scaffolds was determined (Figure 5). As shown in the Figure 5, when compared to the placebo, with a mean CA of 37.85° and 72.61° for 0.2 and 0.4 dl/g PLGA, respectively, the contact angle after the incorporation of ketoprofen was slightly increased. In the present study, both the ketoprofen-eluting scaffolds printed with 0.2 and 0.4 dl/g PLGA showed contact angles of 38.76 ± 1.75° and 83.72 ± 12.42°, respectively, indicating that the surfaces were quite hydrophilic. Similar results were reported previously where the mean CA of pure PLGA with 0.2 and 0.4 dl/g was around 40° and 70°, respectively [43,44]. The values were slightly increased with the incorporation of ketoprofen. This could be due to the hydrophobicity of the drug. However, it is well known that surfaces with contact angles lower than 90° are hydrophilic, whereas surfaces with contact angles above 90° resist wetting. Therefore, ketoprofen-eluting scaffolds could improve the adhesion and proliferation of scaffolds.

### 3.7. In Vitro Release of Ketoprofen from PLGA Scaffolds

#### 3.7.1. Effect of Infill Density

**Figure 6:**
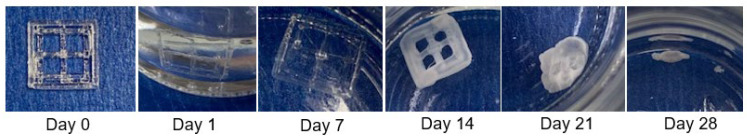
Macroscopic images of the scaffolds at different stages of the in vitro release studies.

**Figure 7:**
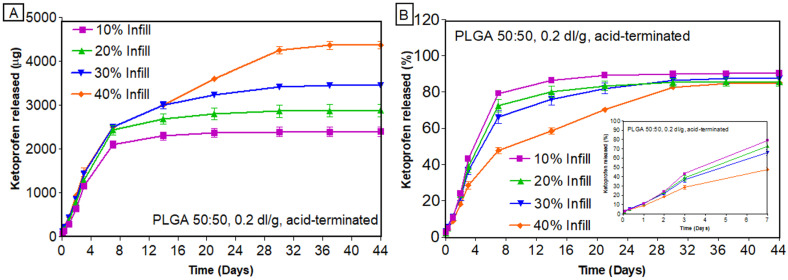
In vitro release of ketoprofen from scaffolds printed with PLGA 0.2 dl/g, with acid-terminated group: (**A**) cumulative amount released (µg); (**B**) cumulative percentage released (n = 3, mean ± standard error of mean (SEM)).

Figure 6 shows macroscopic images of scaffolds at various time points during the in vitro release studies. The first part of this work was focused on determining the impact of 3D printing parameters, such as infill density, on the release of ketoprofen. Ketoprofen scaffolds with two different viscosities of PLGA (0.2 dl/g and 0.4 dl/g) were printed with varying infill densities (10–50%). The in vitro release was dependent on the viscosity of the polymer. Due to the chemistry of PLGA 0.2 dl/g, which has a lower inherent viscosity compared to other PLGAs, it enables faster drug release by erosion. Figure 7 shows the cumulative amount (Figure 7A) and cumulative percentage (Figure 7B) of ketoprofen released from PLGA scaffolds printed with 0.2 dl/g inherent viscosity. It was interesting to observe a drug release of more than 78% in 7 days for 10% infill, whereas 40% infill showed a release of only 47% in 7 days (Figure 7B).

**Figure 8:**
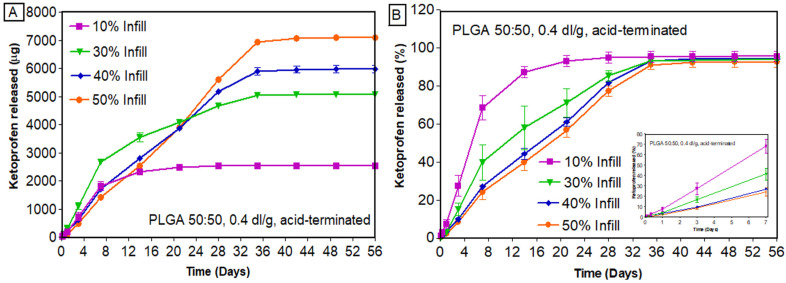
In vitro release of ketoprofen from scaffolds printed with PLGA 0.4 dl/g, acid-terminated: (**A**) cumulative amount released (µg); (**B**) cumulative percentage released (%) (n = 4, mean ± standard error of mean (SEM)).

The trend observed for PLGA 0.2 dl/g was prominent in PLGA 0.4 dl/g (Figure 8), where scaffolds with 10% infill released the drug faster (~67% released in 7 days), and those with 40% infill showed a more sustained release (26% released in 7 days) (Figure 8B). This might be due to the fact that 10% infill has openings across the structure, which would allow the release media to interact with the core during the in vitro release studies. At the same time, the scaffolds with higher infill (50% infill) have less gaps for the release media to interact with the core, which leads to the sustained release of drugs. Previous studies have also reported the effect of infill density on the in vitro release of drugs [45,46]. In a study by Manini et al., higher porosity and surface/volume ratios were observed for scaffolds with lower infill densities, thus providing a higher burst release. As the infill density decreased, the number of printed meshes decreased, and the area of pores created by the printed material became larger [47]. The findings from the current study were also in line with the initial hypothesis of this research that the infill density has a major impact on the drug release behavior and, thereby, the overall performance of the 3D-printed scaffolds. A comparison of the in vitro release of ketoprofen from two different PLGAs printed with different infill densities is provided in Figures S2 and S3. From Figure S3, it is clear that PLGA 0.4 dl/g, due to its higher inherent viscosity, sustained the release of ketoprofen irrespective of the infill densities. As evident from Figure S4, the cumulative percent of ketoprofen remaining in the scaffolds at the end of release was very low, confirming complete and almost 100% drug release from these structures. These differences in the amount released might appear small for drugs like ketoprofen but are massive for drug classes such as anti-hypertensives (atorvastatin, nifedipine) and anticancer drugs (paclitaxel), where the difference of merely 10 mg changes the dosage forms from immediate release to sustained release [48].

#### 3.7.2. Effect of Geometry

**Figure 9:**
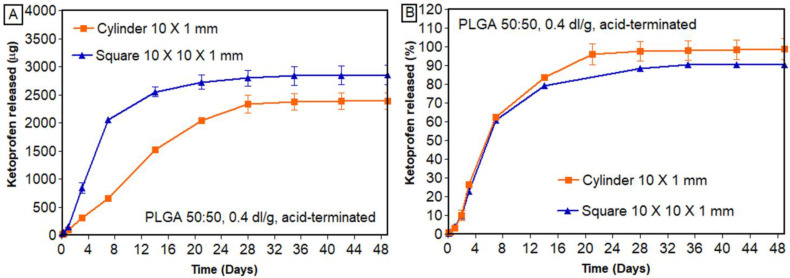
In vitro release of ketoprofen from scaffolds printed with different geometries: (**A**) cumulative amount released; (**B**) cumulative percentage released (n = 3, mean ± standard error of mean).

Scaffolds were printed in the form of cylinders (2.43 mg of drug per implant) and squares (3.66 mg of drug per implant) with similar drug loading (5% DL) and infill density (20% infill). Based on the results (Figure 9), we found that geometrical shape had an effect on the in vitro release of ketoprofen. Structures printed in the form of a cylinder and square released 83.67 ± 0.91% and 79.42 ± 0.35%, respectively, within 14 days.

From the outset, a lower percentage of ketoprofen was released from squares than from cylinders. Even at the end of the release, the difference in the percentage of ketoprofen released remained similar (99% and 91% released from cylinders and squares, respectively). This confirms that the design of a 3D-printed structure has a significant impact on drug release. A similar pattern was observed by Manini et al., where a higher percentage of paliperidone was released from the rings compared to the discs [49]. Goyanes et al. studied the effect of different geometries of paracetamol-loaded 3D-printed tablets (cylinder, pyramid, sphere, cube, and torus). It was observed that geometries with higher surface/volume ratios had a higher percentage of drug released [50]. Similarly, in the current study, cylindrical scaffolds showed higher release of ketoprofen than square-shaped structures.

#### 3.7.3. Effect of Wall Thickness

**Figure 10:**
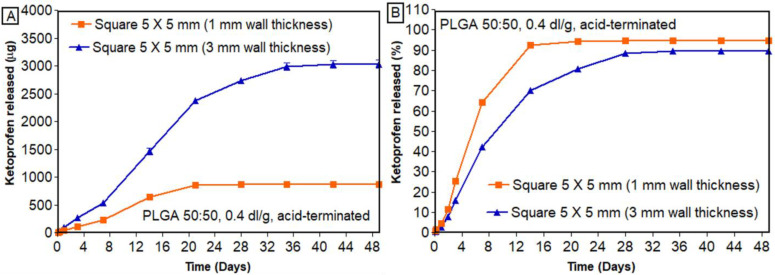
In vitro release of ketoprofen from scaffolds printed with different wall thicknesses: (**A**) cumulative amount released; **(B**) cumulative percentage released (n = 3, mean ± standard error of mean (SEM)).

Figure 10 shows the effect of wall thickness on the in vitro release of ketoprofen. As shown in Figure 10B, scaffolds with smaller wall thickness (1 mm) had a higher percentage of drug release compared to thicker scaffolds (3 mm). The layer thickness has been reported to impact the mechanical strength of 3D-printed structures [51] and influence drug release [47,49,52]. In a study by Yang et al., the impact of shell thickness on the release of ibuprofen was studied. It was reported that 3D-printed tablets with thicker shells had a slower release than thin-shelled tablets [53]. In a similar study by Obeid et al., layer thickness showed a significant impact on the drug dissolution [54]. Similar findings were shown in previous reports where, regardless of the infill pattern, increasing the wall thickness decreased the drug release [53]. This might be due to the fact that increasing the wall thickness increases the density of the material printed, thereby providing sustained release of the drug. Similarly, suitable mechanical properties are necessary characteristics for a scaffold to resist fracture under physiological load. Factors such as mechanical strength and degradation are highly dependent on the infill density and wall thickness, ultimately affecting bone formation [55]. In addition to mechanical stability, another important factor for successful integration into host tissue is the proper development of the vascular network within the scaffold after implantation [56]. This vascular network was successfully developed with a porous scaffold with sufficient wall thickness. Such design can not only provide sufficient mechanical strength but also allow for proper nutrient and waste transport and vascularization. Specifically, structures with an outer shell should be designed to withstand compressive loads, and at the same time, the interior should be porous to allow for nutrient transport and vessel ingrowth [57].

#### 3.7.4. Drug Release Kinetics

The release profiles of all the implantable scaffolds were curve-fitted to mathematical drug release models (Table 3). For both the PLGAs, 0.2 dl/g and PLGA 0.4 dl/g, the release profiles fit well with the Korsmeyer–Peppas model (Figure S5 and Figure S6, respectively). This initial burst release, followed by sustained release, can be attributed to the sink conditions maintained throughout the study and the inherent properties of the polymer where the drug release occurs by slow surface erosion. This mechanism holds true for both the polymers since there was a significant dissolution of the implantable scaffolds, and the key mechanism of release was erosion as the shape of the scaffolds significantly changed over time. This model was used to describe the drug release profiles from polymeric systems and considers both Fickian and non-Fickian drug release mechanisms. This model also considers changes in the shape of the scaffolds over time and hence can explain the release behavior of PLGAs with viscosities 0.2 and 0.4 dl/g. The current study focused only on PLGA-based polymers, where the drug was molecularly dispersed in the polymeric matrix, and the rate-controlling factor for the release was the rate of polymer hydration, which was dependent on the infill density and not the intrinsic property of the drug. Therefore, this study can be used to explain the release behavior of any drug dispersed in such a polymeric matrix.

## 4. Conclusions

In this study, 3D printing technology was utilized to develop biodegradable drug-eluting porous implantable scaffolds for bone regeneration. A good correlation was observed between the infill vs. weight (R^2^ of 0.9877 and 0.9887 for PLGA 0.2 dl/g and 0.4 dl/g, respectively) and between the infill vs. drug loading (R^2^ of 0.9935 and 0.9952 for PLGA 0.2 dl/g and 0.4 dl/g, respectively). DSC thermograms confirm that the polymer successfully stabilized the implant, and the surface recrystallization of the drug was not responsible for the release behavior of the drug. The release data suggested that PLGA scaffolds printed with 0.4 dl/g (26% released in 7 days) successfully controlled the burst release of ketoprofen compared to PLGA scaffolds with 0.2 dl/g inherent viscosity (47% released in 7 days). The release was further dependent on different printing parameters, such as the infill density, geometry, and wall thickness. PLGA scaffolds showed a lower percentage of ketoprofen release from squares (79% released in 14 days) than cylinders (84% released in 14 days). Similarly, wall thickness showed a significant impact on the burst release of ketoprofen. PLGA scaffolds with thicker walls showed lower burst release (42% released in 7 days) compared to thin-layered scaffolds (64% released in 7 days). It can be inferred that the major mechanism of release is the rate of hydration/erosion of the PLGA polymer, which is further dependent on the available surface area. Thus, by adopting thermoplastic extrusion-based 3D printing technology, drug-eluting scaffolds can be satisfactorily manufactured for application in complicated bone repair and restoration. A limitation of the current research is the lack of studies evaluating the biocompatibility and bioactivity of scaffolds. In the future, the bioactivity of ketoprofen-eluting PLGA scaffolds will be assessed both in vitro and in vivo. Cell adhesion, proliferation, and differentiation of human bone mesenchymal stem cells (hBMSCs) will be studied after 1, 3, and 7 days in vitro [58]. Further, these PLGA scaffolds will be implanted into the femoral bone defect of a suitable animal model, and histological analysis of defect regions will be performed to study their effect on bone healing and the formation of connective tissue. The long-term effects of local tissue response after implantation will be studied.

## Figures and Tables

**Table 1:** Optimized 3D printing parameters for the fabrication of PLGA scaffolds.

Printer Parameters	Scaffolds Printed with PLGA 50:50, 0.2 dl/g, Acid-Terminated	Scaffolds Printed with PLGA 50:50, 0.4 dl/g, Acid-Terminated
Printing temperature (°C)	90	120
Print speed (mm/s)	2	2
Nozzle internal diameter (mm)	0.4	0.4
Print head	Thermoplastic	Thermoplastic
Printing technology	Direct powder extrusion	Direct powder extrusion
Print bed temperature (°C)	25	25
Pre and post flow (ms)	50 and 50	50 and 50
First layer height (%)	80	80
Printing pressure (kPa)	200	180
Shape	Rectilinear	Rectilinear
Infill density (%)	10, 20, 30, 40	10, 30, 40, 50
Dimensions (mm)	10 × 10 × 1	10 × 10 × 1
Weight of the scaffolds (mg)	48.49–116.55	51.47–151.03
Amount of drug loading (mg)	2.54–5.34	2.57–6.69
Scaffold thickness (mm)	1	1
Printing pattern	Crosshatch (0°/90°)	Crosshatch (0°/90°)

**Table 2:** Drug content and content uniformity of 3D-printed ketoprofen-eluting implantable scaffolds (PLGA 0.4 dl/g acid-terminated, 10 × 10 × 1 mm, 10% infill) (n = 6 per batch).

Batch	Weight of the Scaffold (mg)	Amount of Ketoprofen (mg)		%Assay (Mean ± SD)
		Theoretical	Actual	
Batch 1	52.55 ± 7.30	2.39 ± 0.42	2.34 ± 0.32	98.76 ± 5.89
Batch 2	52.80 ± 2.06	2.67 ± 0.10	2.68 ± 0.10	100.46 ± 3.19
Batch 3	48.90 ± 8.34	2.91 ± 0.08	2.92 ± 0.14	100.29 ± 2.98

**Table 3:** Different mathematical models for ketoprofen-eluting PLGA scaffolds.

Factors Studied	Percent Infill	Zero Order (R^2^)	First Order (R^2^)	Higuchi Order (R^2^)	Hixson–Crowell (R^2^)	Korsmeyer–Peppas *		
						n	K	R^2^
PLGA 50:50, 0.2 dl/g, acid-terminated	10%	0.6484	0.485	0.829	0.555	0.609 ± 0.025	0.288 ± 0.071	0.933 ± 0.007
	20%	0.672	0.485	0.849	0.566	0.613 ± 0.036	0.253 ± 0.077	0.941 ± 0.015
	30%	0.734	0.533	0.895	0.615	0.575 ± 0.051	0.340 ± 0.154	0.957 ± 0.008
	40%	0.860	0.595	0.970	0.706	0.607 ± 0.011	0.196 ± 0.049	0.978 ± 0.007
PLGA 50:50, 0.4 dl/g, acid-terminated	10%	0.660	0.495	0.916	0.567	0.705 ± 0.099	0.032 ± 0.002	0.947 ± 0.024
	30%	0.839	0.566	0.898	0.687	0.810 ± 0.091	0.411 ± 0.259	0.976 ± 0.011
	40%	0.906	0.649	0.826	0.778	0.849 ± 0.045	0.561 ± 0.138	0.990 ± 0.001
	50%	0.918	0.666	0.809	0.794	0.880 ± 0.052	0.678 ± 0.321	0.985 ± 0.003

* The first 60% of drug release data were fitted into the model.

## Data Availability

No new data were created or analyzed in this study. Data sharing is not applicable to this article.

## Supplementary Materials

The following supporting information can be downloaded at: https://www.mdpi.com/article/10.3390/bioengineering11030259/s1, Table S1. Solubility of ketoprofen in different pharmaceutical solvents at 25 °C (n = 3, mean ± SD). Table S2. Physical properties of PLGA 50:50 polymer. Figure S1. Compression strength of 3D-printed PLGA and ketoprofen + PLGA scaffolds printed with different infill densities (n = 3, mean ± standard error of mean). Figure S2. Comparison of in vitro release of ketoprofen (cumulative amount released) from discs printed with PLGA 0.2 dl/g and 0.4 dl/g and with different infill densities (n = 3, mean ± standard error of mean (SEM)). Figure S3. Comparison of in vitro release of ketoprofen (cumulative percent released) from discs printed with PLGA 0.2 dl/g and 0.4 dl/g and with different infill densities (n = 3, mean ± standard error of mean (SEM)). Figure S4. Cumulative percentage of ketoprofen remaining in discs printed with different infill densities: (A) PLGA 50:50, 0.2 dl/g, acid-terminated; (B) PLGA 50:50, 0.4 dl/g, acid-terminated (n = 3, mean ± standard error of mean (SEM)). Figure S5. Various mathematical models for the in vitro release of ketoprofen from discs printed with PLGA 50:50, 0.2 dl/g, acid-terminated polymer at different infill densities. Figure S6. Various mathematical models for the in vitro release of ketoprofen from discs printed with PLGA 50:50, 0.4 dl/g, acid-terminated polymer at different infill densities.
