# Supplementary material for: Three-Dimensional Printing of Drug-Eluting Implantable PLGA Scaffolds for Bone Regeneration
Source: Bioengineering (Basel). 2024 Mar 6;11(3):259. doi: 10.3390/bioengineering11030259 (PMC10967843; doi:10.3390/bioengineering11030259)
Supplement: Supplementary file 1 [file bioengineering-11-00259-s001.zip › bioengineering-2789099-supplementary.pdf]

bioengineering

MDPI

Supplementary Materials

Three-Dimensional Printing of Drug-Eluting Implantable PLGA Scaffolds for Bone Regeneration

Manjusha Annaji [1], Nur Mita [1,2], Ishwor Poudel [1], Sai H. S. Boddu [3,4], Oladiran Fasina [5] and R. Jayachandra Babu [1,*]

1 Department of Drug Discovery and Development, Harrison College of Pharmacy, Auburn University, Auburn, AL 36849, USA; mza0153@auburn.edu (M.A.); nzm0076@auburn.edu or mita@farmasi.unmul.ac.id (N.M.); izp0018@auburn.edu (I.P.)
2 Faculty of Pharmacy, Mulawarman University, Samarinda, Kalimantan Timur 75119, Indonesia
3 Department of Pharmaceutical Sciences, College of Pharmacy and Health Sciences, Ajman University, Ajman P.O. Box 346, United Arab Emirates; s.boddu@ajman.ac.ae
4 Center of Medical and Bio-Allied Health Sciences Research, Ajman University, Ajman P.O. Box 346, United Arab Emirates
5 Department of Biosystems Engineering, Samuel Ginn College of Engineering, Auburn University, Auburn, AL 36849, USA; fasinoo@auburn.edu
* Correspondence: ramapjb@auburn.edu

Table S1. Solubility of ketoprofen in different pharmaceutical solvents at 25 °C (n = 3, mean ± SD).

Solvent	Solubility of Ketoprofen (mg/mL)
PBS pH 7.4	0.404 ± 0.21
Water	0.135 ± 0.38
PBS pH 7.4: Ethanol (9:1% v/v)	0.405 ± 0.16
PBS pH 7.4: Ethanol (8:2% v/v)	0.415 ± 0.04
PBS pH 7.4: Methanol (9:1% v/v)	0.399 ± 1.32

Table S2. Physical properties of PLGA 50:50 polymer.

Polymer	Glass transition temperature (°C)	Tensile strength (MPa)	Elongation (%)	Modulus (Pa)	Biodegradation time (Months)
PLGA 50:50	30–45	40–55	3–10	140–280	1–2

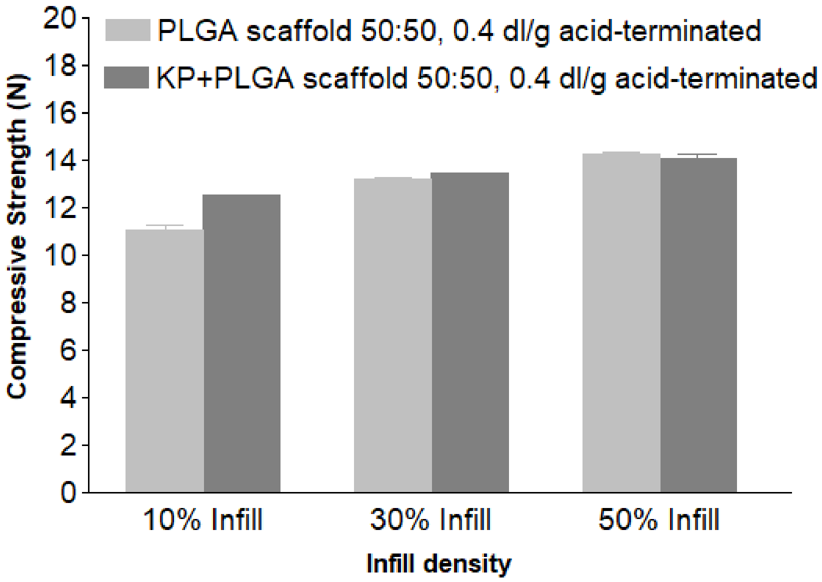

Figure S1. Compression strength of 3D printed PLGA and Ketoprofen + PLGA scaffolds printed with different infill densities (n = 3, mean ± standard error of mean).

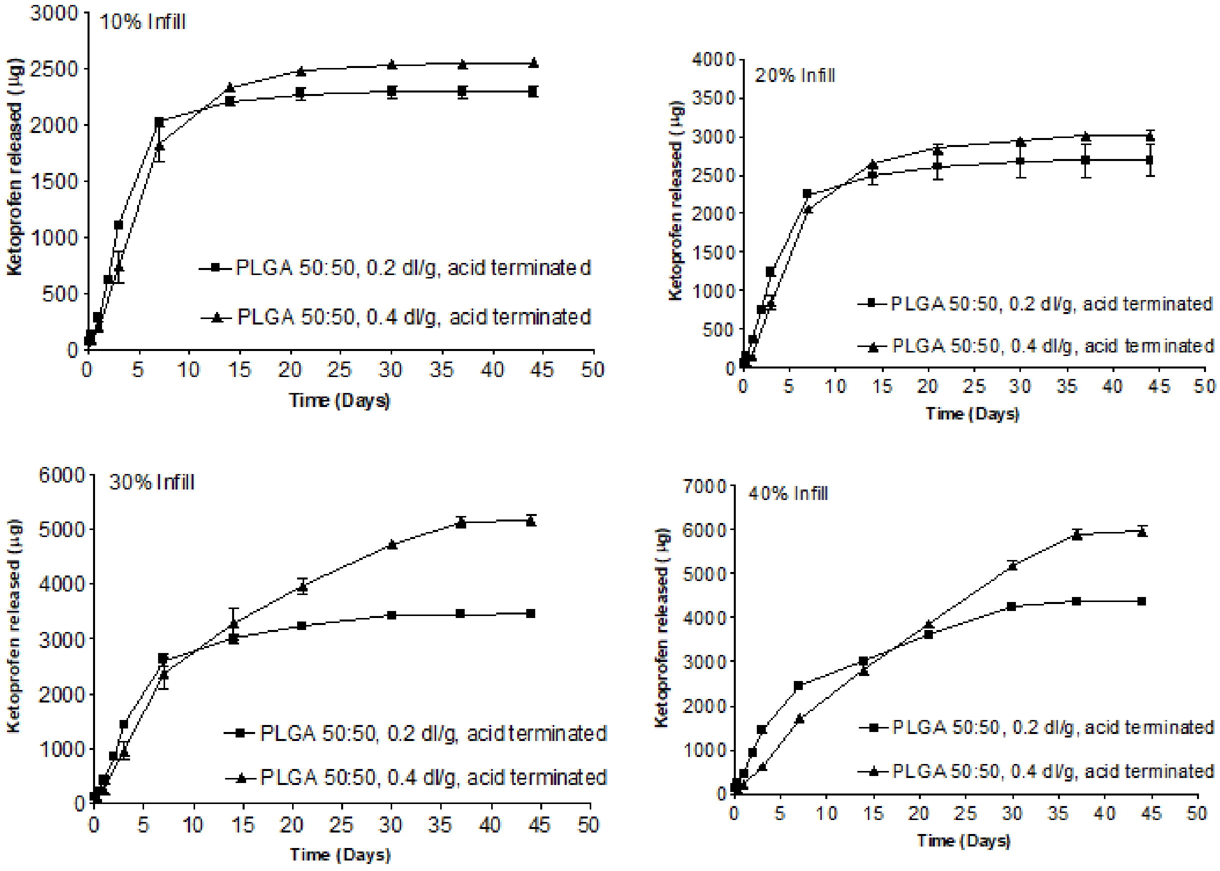

Figure S2. Comparison of in vitro release of ketoprofen (cumulative amount released) from discs printed with PLGA 0.2 dl/g and 0.4 dl/g and with different infill densities (n = 3, mean ± standard error of mean (SEM)).

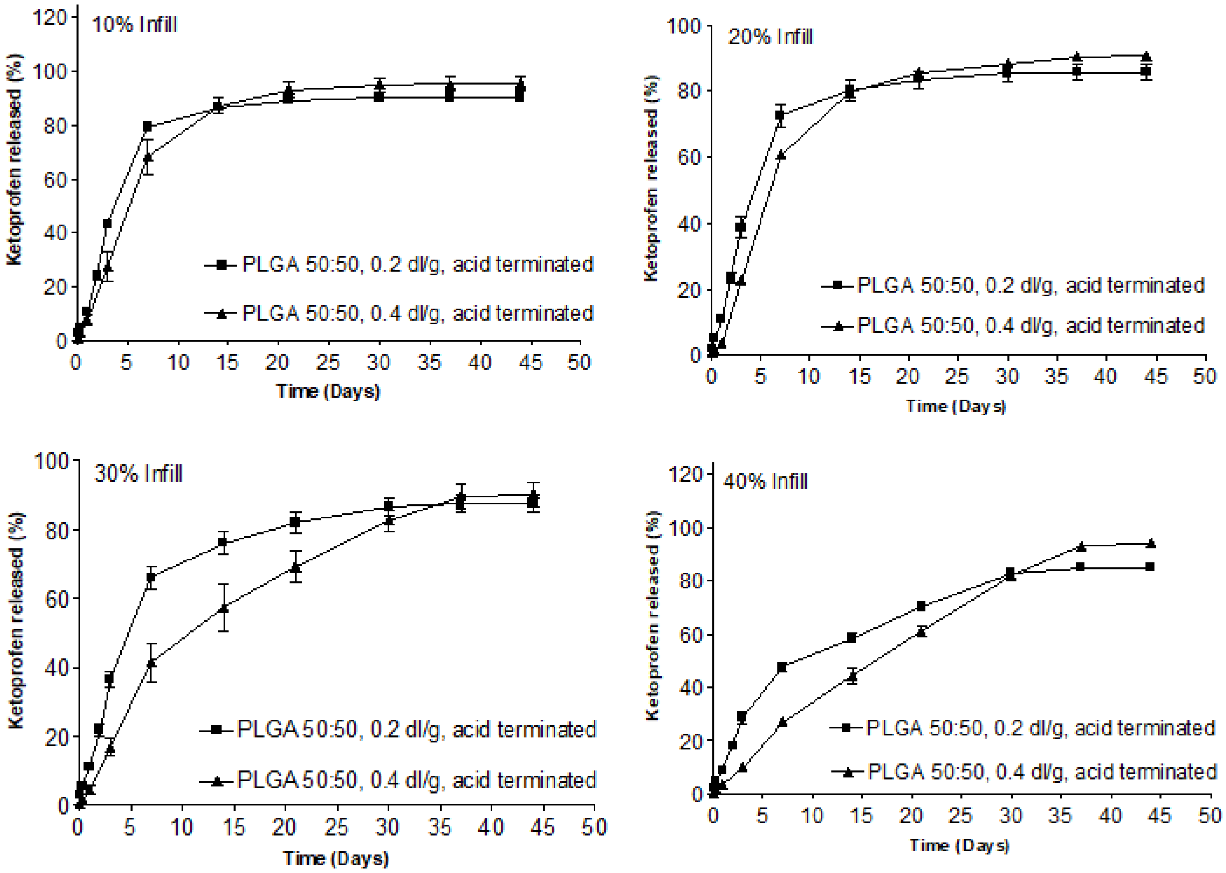

Figure S3. Comparison of in vitro release of ketoprofen (cumulative percent released) from discs printed with PLGA 0.2 dl/g and 0.4 dl/g and with different infill densities (n = 3, mean ± standard error of mean (SEM)).

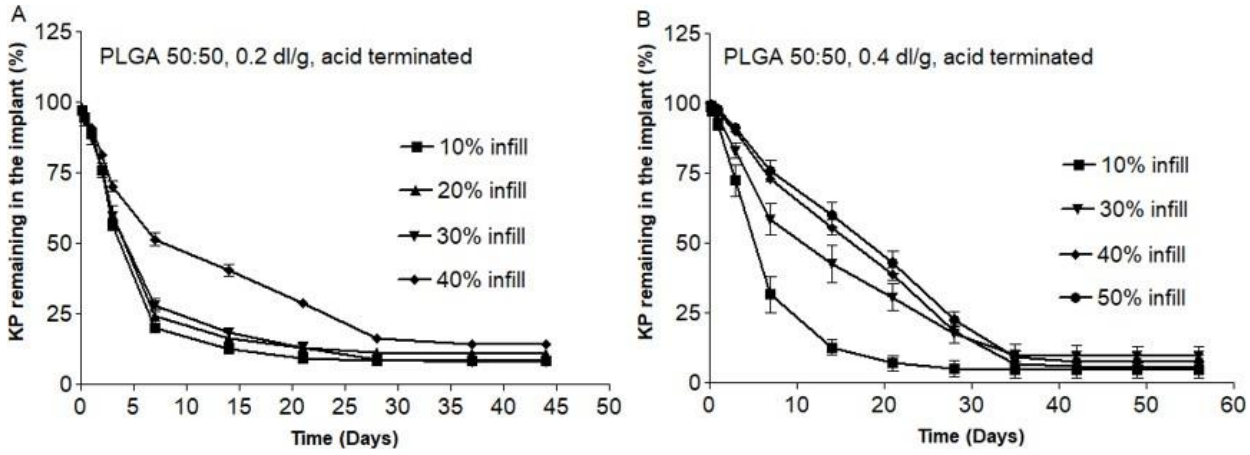

Figure S4. Cumulative percentage of ketoprofen remaining in the discs printed with different infill densities (**A**) PLGA 50:50, 0.2 dl/g, acid-terminated; (**B**) PLGA 50:50, 0.4 dl/g, acid-terminated (n = 3, mean ± standard error of mean (SEM)).

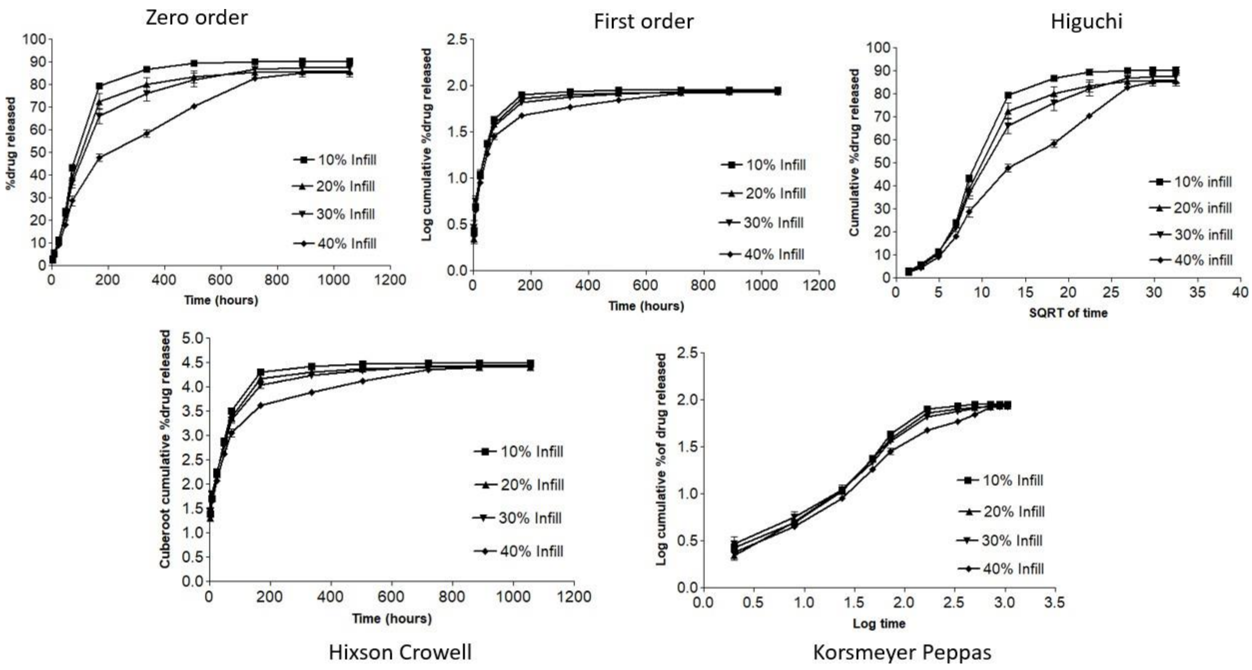

Figure S5. Various mathematical models for the in vitro release of ketoprofen from discs printed with PLGA 50:50, 0.2 dl/g, acid terminated polymer at different infill densities.

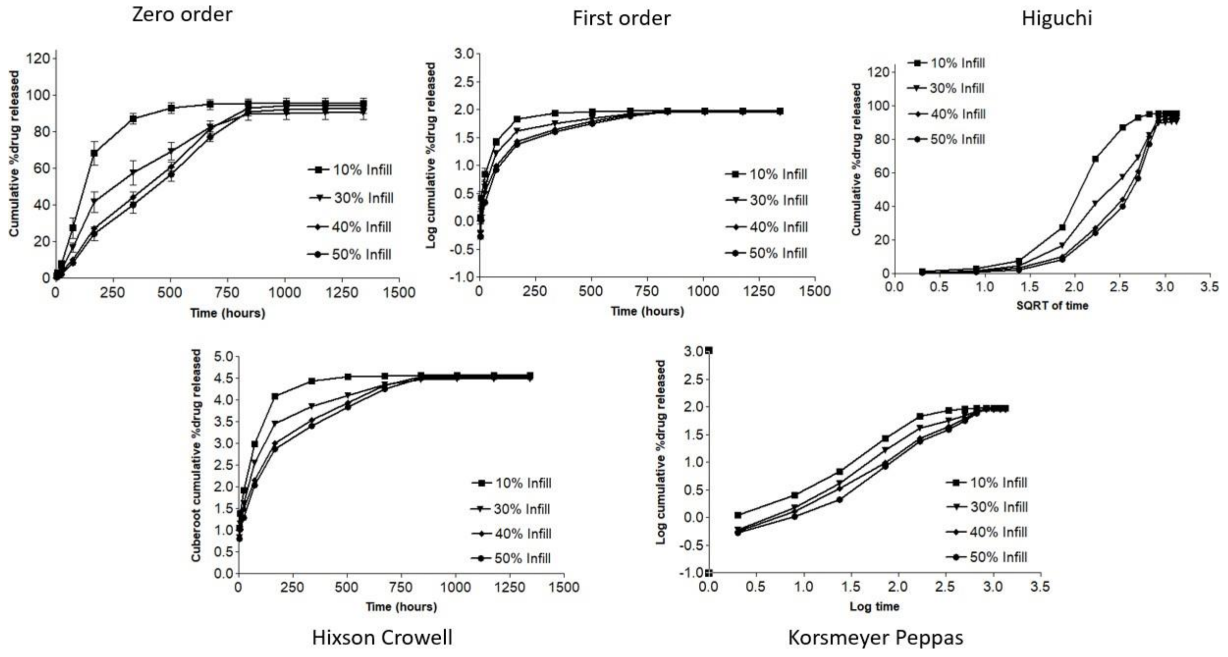

Figure S6. Various mathematical models for the in vitro release of ketoprofen from discs printed with PLGA 50:50, 0.4 dl/g, acid terminated polymer at different infill densities.
